# Chimerism Monitoring Techniques after Hematopoietic Stem Cell Transplantation: An Overview of the Last 15 Years of Innovations

**DOI:** 10.3390/diagnostics11040621

**Published:** 2021-03-30

**Authors:** Pamela Tozzo, Arianna Delicati, Renato Zambello, Luciana Caenazzo

**Affiliations:** 1 Department of Molecular Medicine, Laboratory of Forensic Genetics, University of Padova, 35121 Padova, Italy; arianna.delicati@studenti.univr.it (A.D.); luciana.caenazzo@unipd.it (L.C.); 2 Department of Medicine (DIMED), Hematology and Clinical Immunology Section, Padova University School of Medicine, 35121 Padova, Italy; r.zambello@unipd.it

**Keywords:** chimerism analysis, hematopoietic stem cell transplantation, STR-PCR, qPCR, dPCR, NGS techniques

## Abstract

Chimerism analysis is a well-established method for monitoring the state of hematopoietic stem cell transplantation (HSCT) over time by analyzing peripheral blood or bone marrow samples of the recipient in several malignant and non-malignant hematologic diseases. From a clinical point of view, a continuous monitoring is fundamental for an effective early therapeutic intervention. This paper provides a comparative overview of the main molecular biology techniques which can be used to study chimerism after bone marrow transplantation, focusing on their advantages and disadvantages. According to the examined literature, short tandem repeats (STR) analysis through simple PCR coupled with capillary electrophoresis (STR-PCR) is the most powerful method which guarantees a high power of differentiation between different individuals. However, other methods such as real-time quantitative PCR (qPCR), digital PCR (dPCR), and next-generation sequencing (NGS) technology were developed to overcome the technical limits of STR-PCR. In particular, these other techniques guarantee a higher sensitivity, which allows for the detection of chimerism at an earlier stage, hence expanding the window for therapeutic intervention. After a comparative evaluation of the various techniques, it seems clear that STR-PCR still remains the gold standard option for chimerism study, even if it is likely that both dPCR and NGS could supplement or even replace the common methods of STR analysis.

## 1. Introduction

The word chimerism derives from the term “chimera”, which is a monster from Greek mythology that is part lion, goat, and serpent [1]. From a medical point of view, the word chimera is used to define an individual in whom cell populations from genetically different individuals coexist. This phenomenon can occur either spontaneously, such as in the cases of feto-fetal transfer [2] or feto-maternal transmission [3], or artificially in the case of allogeneic hematopoietic stem cell transplantation (HSCT) [4,5,6,7].

Currently, allogeneic HSCT is an efficient curative approach for different hematologic diseases, but it can also be characterized by various complications, such as toxicity related to treatment, infections, recurrence of the disease, and immunological reactions including rejection of the transplantation by the recipient and graft-versus-host disease (GVHD) [8,9,10]. The success of allogeneic HSCT is evaluated by chimerism analysis, through which the monitoring of the relative amount of living donor cells and residual recipient cells is performed in samples of peripheral blood or bone marrow; what is clinically useful is not the single determination, but the monitoring over time of the dynamics or the changes in the percentages of donor and recipient cells between different time-points [11]. Chimerism analysis is performed to evaluate the engraft status of the transplanted cells and the eventual recurrence of the disease. In addition, minimal residual disease (MRD) analysis can also provide significant prognostic information regarding relapse of disease, thus influencing clinical decisions [12].

Peripheral blood (PB) and bone marrow (BM) are the preferred biological sources used to define post-transplantation chimerism. Sometimes, lineage specific analysis is also performed to evaluate the status of the chimerism in a subset of hematopoietic cells, increasing its sensitivity but requiring extra steps of purification and isolation, thus increasing its cost and time [13,14]. Chimerism status is categorized as follows: “complete chimerism” (CC) when only the donor’s genotype is detected; “mixed-chimerism” (MC) when the donor and the recipient’s genotypes are detected together and the recipient’s genotype is at least 1%; “micro-chimerism” (Mc) when the recipient’s genotype is lower than 1%; and “split chimerism” where there are one or more recipient and donor lineages at the same time [5,15,16,17]. MC is then further divided into transient MC, stable MC, and progressive MC [18,19].

The clinical significance of chimerism status remains controversial. Different studies suggest a different relation between CC, MC, GVHD, and relapse [4,12,15,20]. In particular, the presence of progressive MC (usually an increase of 5% of recipient DNA) seems to indicate the recurrence of the disease differently from transient or stable MC. Interestingly, an early CC has been reported to be associated with acute GVHD occurrence [19,21,22,23,24]. Moreover, it seems clear that MC could be considered a high-risk factor for relapse. With the advancement of molecular technology, studies investigating MC have emerged, defined as highly sensitive techniques to analyze chimerism at levels below a 1% detection limit. Therefore, the early detection of MC might lead to a beneficial effect thanks to a rapid and appropriate medical intervention, which would reduce the probability of progression to the high-risk category of MC [16]. However, some scholars disagree with this last theory [25,26,27], believing that very high sensitivity might negatively influence the results of chimerism analysis due to the “contamination” caused by the recipient’s cells (skin, endothelium) increasing the false-positive rate [26,27]. Furthermore, other variables conditioning the analysis are represented by the definition of cut-off for MC, affecting the time at which a sample becomes positive, and the kinetics of achieving CDC below 1% in the early post HSCT [28]. Overall, a major limitation of MC analysis, as with chimerism analysis in general, is the consistent report of patients with relapse without prior detection of MC as well as patients with MC that maintain the complete remission. Nevertheless, it is commonly agreed that a continuous chimerism monitoring should be performed in order to favor a timely intervention to expand the window for intervention, thus reducing the negative outcome [22,27,28,29,30].

Currently, chimerism analysis is commonly performed evaluating short tandem repeat (STR) profiles. However, other DNA polymorphisms such as variable number of tandem repeats (VNTR), single nucleotide polymorphisms (SNPs), or insertion/deletion polymorphisms (Indel) can be used. The main reason STRs are more suitable for chimerism analysis compared with other polymorphisms is their higher informativity rate [31,32,33,34]. Nevertheless, a careful selection of STR alleles is required to select the most suitable STR for chimerism monitoring considering all the variables which can affect the analysis [18,35,36].

Originally, chimerism studies were based on phenotyping and cytogenetic techniques, such as fluorescence in situ hybridization (FISH) [37,38,39], which can be applied only to sex-mismatched donor recipient pairs as a limiting factor for routine application [40]. Subsequently, polymerase chain reaction (PCR) and fragments analysis were adopted to study VNTRs or STRs [38,39]. In particular, STR analysis is considered the gold standard technology for chimerism monitoring after allogeneic HSCT, as specified in the EuroChimerism Consortium guidelines [38,41,42]. Nevertheless, a sensitivity of around 1% of STR-PCR analysis may represent an obstacle for timely intervention [12,17,38,43]. In 2002, Alizadeh et al. [44] proved the higher sensitivity of quantitative real-time PCR (qPCR) applied to SNPs in comparison with the common STR-PCR analysis. Additional improvements in terms of sensitivity were observed when qPCR was applied to Indel polymorphisms [40]. Two recently developed technologies, namely digital PCR (dPCR) and next generation sequencing (NGS), are deemed to represent the future of chimerism analysis thanks to their high sensitivity, precision, and breadth of application [38,45].

Given that chimerism analysis plays a crucial role in monitoring the health of patients after allogeneic HSCT, the emergence of innovative and powerful techniques able to measure the levels of donor and recipient cells (also when present in scarce amounts) is something of great interest. However, the various techniques have different features, which can make them more or less appropriate to chimerism monitoring depending on the specific circumstances of the precise case being examined. This review aims to present and evaluate the current knowledge regarding the available molecular biology techniques for the detection of chimerism. As noted, other techniques out of the molecular biology field, such as flow cytometry, have been proposed as alternative tools for chimerism study [46,47]. However, the analysis of these alternative methods goes beyond the scope of this manuscript.

## 2. Materials and Methods

This review was performed in accordance with the Preferred Reporting Items for Systemic Reviews and Meta-Analyses (PRISMA) Guidelines [48].

The articles to be analyzed were selected from PubMed database according to two different queries: “((chimerism) AND (transplantation)) AND (((STR) OR (SNP)) OR (Indel))” and “((chimerism) AND (transplantation)) AND ((PCR) OR (sequencing) OR (electrophoresis))”. A total of 1111 works were identified.

Duplicates were then removed, and different inclusion criteria were applied using specific PubMed filters to start the screening process: (1) publication period from 2005 to 2021; (2) English language; (3) availability of abstract and full text.

Subsequently, the process continued through the screening of titles and abstracts which was followed by the evaluation of the full text of those articles not excluded on the basis of the latter. In cases of doubt, the consensus opinions of the research supervisors were solicited.

A total of 439 articles were thus examined on the basis of title and abstract. Papers regarding solid tumors, specific class of cells, non-human organisms, fetal–maternal chimerism, therapeutic treatments to fight chimerism, or including content not relevant to the aim of the review were excluded during this phase of screening, ending up with 112 works which were subsequently examined in their full texts to be declared eligible.

In this last phase, articles were excluded if considered irrelevant or redundant in content. Eventually, a total of 32 studies were selected.

**Figure 1:**
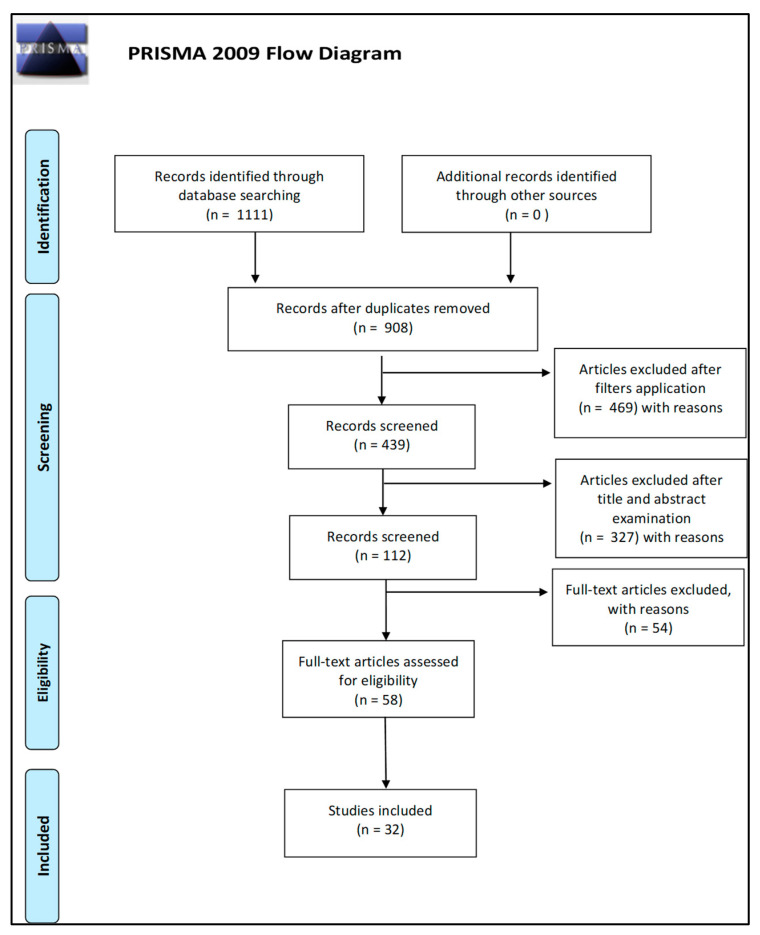
Preferred Reporting Items for Systemic Reviews and Meta-Analyses (PRISMA) 2009 flow diagram.

The number of articles excluded or included was registered and reported in the following PRISMA flowchart (Figure 1).

## 3. Results

This review presents the current knowledge regarding the available molecular biology techniques which are commonly used or which show enormous potential for routine analysis of chimerism. In particular, the focus is on the main advantages and disadvantages of each technique. In order to provide the reader with a better understanding, we grouped the selected studies into the following four categories: (a) chimerism monitoring through STR-PCR; (b) chimerism monitoring through qPCR; (c) chimerism monitoring through dPCR; and (d) chimerism monitoring through NGS.

In addition to the description of each category, the key aspects are summarized at the end of the chapter in Table 1 to provide an overview of the most common and recent molecular biology techniques used for chimerism monitoring after allogeneic HSCT.

### 3.1. Chimerism Monitoring through STR-PCR

Many scholars consider the analysis of STR polymorphisms to be the gold standard technology for chimerism monitoring after allogeneic HSCT [17,38]. According to Andrikovics et al. [31], STRs (known also as microsatellites) are composed of a half dozen or several dozen tandem repeated domains made up of 2–6 nucleotides and are highly useful both in forensic and diagnostic fields.

STR-PCR analysis consists of the simultaneous amplification through PCR of some selected STR loci (multiplex-STRs) combined with DNA fragment analysis performed through capillary electrophoresis, as suggested by Mika et al. [49]. Frankfurt et al. [50] stated that, in the case of chimerism monitoring, pretransplant samples are used to select recipient (and donor) diagnostic alleles (peaks) for post-transplant chimerism analysis. In fact, one of the main advantages of STR-PCR is that both donor and recipient markers are amplified in the same PCR tube, which allows accurate quantification and avoids the need for pre-transplant sample for comparison/quantification. Moreover, they considered that the result of this analysis is the relative ratio of the amount of donor and recipient cells at that particular time point [50]. However, even if pre-transplantation donor and recipient DNA are not available, several solutions for STR-PCR follow-up exist. Indeed, Navarro-Bailón et al. [34] asserted that donor or recipient genotypes can be obtained through successive DNA extraction and analysis from buccal swab or from other biological sources. Moreover, they also stated that if only one between the donor or the recipient is available for DNA extraction, STR variability (which implies a low probability that donor and recipient share the same STR alleles) allows for the identification of the amount of recipient cells after allogeneic HSCT [34]. Li et al. [51] pointed out that, after the transplantation, the collection of recipient biological material through buccal swab may represent a problem given that a buccal swab after allogeneic HSCT can manifest MC. However, for Navarro-Bailón et al. [34], when donor STR profile is available, it is possible to ignore those peaks of the recipient’s buccal swab electropherogram corresponding to donor alleles, permitting the ready identification of those of the recipient. Furthermore, Li et al. [51] also demonstrated that the recipient’s profile could be easily obtained from other biological material, such as hair follicles or semen, for which the genome is 100% recipient type.

As stated by Kristt et al. [29], STR-PCR has the ability to differentiate between individuals, even in the case of multi-donor allogeneic HSCTs, when there is a higher probability of having shared alleles. However, some authors have suggested that undertaking a careful process of STR-marker selection is crucial in all the cases of chimerism monitoring in order to guarantee an accurate and reliable result. This selection should be performed to choose the most suitable STR for the chimerism monitoring considering all the variables (both instrumental and biological) that can affect the analysis [18,35,52]. For instance, many scholars agree that STR markers should be excluded from the chimerism calculation when the recipient’s stutter peak coincides with one of the donor’s peaks or vice versa in order to avoid interference in chimerism quantification. This is also true for markers which are shared between the donor and the recipient, given the impossibility of identifying the origin of the STR alleles [18,35,36,37,41]. As claimed by Kristt et al. [28] and Navarro-Bailón et al. [34], this latter circumstance is more common in the case of transplantation occurring between related donor–recipient pairs than in the case of transplantation between unrelated pairs. In accordance with Lion et al. [41], STR markers differing significantly in size between the donor and the recipient should also be excluded from the calculation, because their different length may result in preferential amplification of the smaller allele, which in turn may cause allelic imbalance and errors in the interpretation from a quantitative point of view. Moreover, Gineikiene et al. [18] observed that other problems such as the presence of pull-up peaks or off-scale peaks can make it difficult to determine the exact area of the peaks. They suggested that, to overcome this problem, the analysis could be performed a second time using a lower amount of PCR product loaded into the analyser, decreasing the injection time, or using a lower amount of DNA in the amplification phase [18]. Eventually, both Gineikiene et al. [18] and Clark et al. [36] suggested that all these potential errors influencing the quantification of each allele and, in turn, their peak area affect the results of the chimerism analysis given that the chimerism percentage in a post-transplantation sample is calculated by dividing the recipient’s peak area by the sum of recipient and donor’s peaks area at the chosen position.

The STRs are also selected based on their informative power, differentiation rate, variability, and heterozygosity. However, the main feature which makes them suitable for chimerism analysis is that they are multi-allelic polymorphisms with a higher informative rate in comparison with the other types of polymorphisms, as suggested by some authors [31,34]. Moreover, Vives et al. [47] and Frankfurt et al. [50] stated that the analysis of the STRs in multiplex permit for the consumption of a limited amount of DNA as well as the simultaneous processing of both donor and recipient markers in the same reaction tube.

In general, chimerism monitoring through this technique guarantees the detection of MC with a sensitivity of 1%. However, it seems that this sensitivity of 1% attributed to STRs is sometimes not sufficient to allow early intervention [18,26,43,53]. With the purpose of overcoming this limit, it is possible to analyse the different types of cells previously isolated. In particular, even if this particular topic is beyond the scope of this review, some works proved that lineage-specific analysis may reach a reasonable sensitivity to detect a threatening relapse and enable therapeutic interventions at an earlier time point. Nevertheless, this strategy incurs higher costs and greater time due to the extra steps of cell isolation and purification [34,40].

Tseng et al. [54] highlighted the phenomenon of microsatellite instability (MSI) which entails the alteration in length of the STRs causing the appearance of an extra allele or of a new allele. For this reason, these authors [54] and Han et al. [35] believe that more than one locus should be used to estimate the chimerism status. More than one locus, and at least two of them, should be used for chimerism analysis whenever possible, and quantification should be derived from the mean of the results with the different individual markers. In particular, the MSI should be considered during the entire process of chimerism monitoring in order not to confound the analysis by excluding the STRs commonly affected by losses or gains of repeats. 

Han et al. [35] performed the analysis of STR genotypes of 1249 donor–recipient pairs concluding that the use of more than one locus improves the reliability of the chimerism analysis, especially in the case of related pairs for which the number of loci to consider is higher than the one of unrelated pairs.

Therefore, given all the mentioned features making it a simple, universal, and cheap technique, many authors agree that STR-PCR represents a valid tool to guarantee rapid and reliable chimerism monitoring [18,34,50,55].

### 3.2. Chimerism Monitoring through qPCR

Andrikovics et al. described the qPCR as an easy, sensitive, and fast technique characterized by a rapid turnaround time which allows for the detection and the quantification of the DNA in a given sample [31]. It consists of the simultaneous amplification and quantification of some DNA sequences of interest (e.g., SNP or Indel markers in the case of chimerism monitoring) through the detection of amplicons in real time, which is made possible by exploiting fluorescence emission. In particular, two different methods are commonly adopted. The first is based on the relationship between a quencher fluorescent dye and a reporter fluorescent dye. Among the reagents contained in the mix of the reaction, there is a probe which contains both fluorophores and is designed to pair with the target DNA sequence to amplify. If this probe is intact, the fluorophores are close to each other and, following a specific laser excitation, the quenching occurs. Therefore, the fluorescence released by the reporter is absorbed by the quencher. Instead, during 5′→3′ extension phases of qPCR, the quenching effect is not possible because the probe is cleaved by the DNA polymerase, and the fluorophores are released and move away from each other. At this point, the fluorescence of the reporter is emitted and detected as a signal. The second method is based on the fluorescence resonance energy transfer (FRET) phenomenon. Among the reagents contained in the mix of the reaction, there are two probes, each of them containing one fluorophore. The two probes are designed to pair with two close regions of the target DNA sequence to amplify. If the probes are bound to the target DNA, FRET occurs after a specific laser excitation, and the fluorescence emitted by the first fluorophore is absorbed by the second one, which emits its fluorescence signal. Instead, during 5′→3′ extension phases of qPCR, the probes are detached from the target DNA sequence by DNA polymerase, and the fluorophores move away from each other, therefore the FRET does not occur, and the fluorescence of the second fluorophore is not released. In any case, the fluorescent emission is proportional to the amount of DNA in the sample in both cases.

Different studies suggest that qPCR could be a suitable technique for chimerism monitoring after allogeneic HSCT given that it has a sensitivity of 0.01–0.1% [31,38,47,55]. According to Bach et al. [7] and Jiménez-Velasco et al. [40], this enhanced sensitivity may result in earlier diagnosis of disease relapse, thus increasing the window for therapeutic interventions. However, Navarro-Bailón et al. [34] also noted that this enhanced sensitivity may increase the number of false positive results. For instance, Stahl et al. [26] proposed that chimerism detected after an allogeneic HSCT could be falsely due to “contamination” by recipient’s cells of skin or endothelium. In any case, for some authors, the effect of the higher sensitivity on the clinical outcome of the patient is still under evaluation [49,50,52].

Some authors stated that, in case of chimerism monitoring, qPCR can be used in combination with SNP or Indel polymorphisms given that STRs are repetitive DNA sequences which are not suitable for allele-specific qPCR [9,18,55]. Conversely, different authors have also underlined that SNPs and Indels are bi-allelic sequence polymorphisms which result, respectively, from independent mutational events and from insertion or deletion of short sequences in the genome. However, they consider that bi-allelic polymorphisms have a low capacity of differentiation between individuals. Therefore, a huge panel of biallelic markers should be considered to achieve an acceptable informative rate in chimerism monitoring [9,18,31].

There are important technical limits which can affect the application of qPCR to the study of chimerism. First of all, in qPCR, the assessment of chimerism is performed using specific markers that should be previously adapted to the recipient–donor couple [56]. For instance, Navarro-Bailón et al. [34] and Tyler et al. [55] highlighted that the bi-allelic markers analyzed through qPCR do not always guarantee that a distinction will be made between recipients and donors in the case of transplantation from multiple donors (e.g., in the case of double-donor transplantation, SNP and Indel markers are not informative if the same alleles are present in all three individuals). Moreover, the same authors have claimed that the experimental procedure of qPCR requires a high amount of input DNA because pre-transplantation DNA samples of both donor and recipient are required to generate calibration curves for each assay. This amount of input DNA may represent a limit given that the amount of sample required could not be available to support long-term monitoring [34,55]. Various solutions have been proposed by different authors to address this problem: Tyler et al. [55] suggested to calculate a reference value (ΔCq) to normalize the post-transplantation DNA amount; Bach et al. [7] demonstrated that the use of a lower amount of DNA to perform qPCR analysis does not affect PCR efficiency; Frankfurt et al. [50] suggested adopting the last available samples as a reference for the following analysis (pre-transplantation samples can be used as a reference for the first post-transplantation analysis, the first post-transplantation sample can be used for the second post-transplantation analysis, etc.). Besides these three options, Bach et al. [7] proposed a fourth alternative solution which consists of improving DNA extraction and purification in order to start the process of amplification with a higher amount of DNA. This optimization could lead to an increase in the sensitivity of chimerism monitoring after allogeneic HSCT, but, as stated by Masmas et al. [56], it could also increase the amount of post-transplantation DNA, resulting in an increased level of both background signal and PCR inhibition. Remarkably, these latter effects could interfere with the analysis and, in particular, with the detection of MC [56]. Nevertheless, as described by Navarro-Bailón et al. [34], recipient and donor pre-transplantation samples may sometimes not be available. In this case, the collection of donor and recipient buccal swabs could not be a solution as it is for STR-PCR due to the high sensitivity of the qPCR. They claimed that this sensitivity could indeed lead to detecting an MC in the recipient’s buccal swab rather than an SNP/Indel recipient’s genotype given that, as demonstrated by Li et al. [51], chimerism may be detected in a certain percentage in a recipient’s buccal swab after the HSCT. Moreover, Navarro-Bailón et al. [34] sustained that, given that the markers are bi-allelic, a simple comparison between the two buccal swabs could not ensure the correct typing of the individuals. This could significantly affect the follow-up of qPCR chimerism analysis due to the previously mentioned fact that the recipient’s pre-transplantation sample is required to generate calibration curves in each assay. However, Li et al. [51] suggested that other biological sources (e.g., hair or semen) which maintain 100% of a recipient’s cells could be suitable in detecting a recipient’s pre-transplantation genotype, allowing for the partial overcoming of its lack thereof.

In addition, some authors stated that the cost of a qPCR reaction for chimerism monitoring can be high because qPCR entails a singleplex analysis while the analysis of several bi-allelic markers may be required for the aim of chimerism monitoring, thus requiring a great amount of both DNA sample and reagents [34,50,57]. Nadvornikova et al. [57] recently developed a first-of-its-kind multiplex qPCR which should guarantee an increase in throughput and reliability as well as a decrease in errors and costs in comparison with the current singleplex qPCR technique. However, for Frankfurt et al. [50], the informative rate remains limited even in the case of multiplex qPCR analysis given that the high cost of reagents does not permit the simultaneous analysis of too many markers per assay.

Bach et al. [7] conducted an analysis of Indel markers in 16 allogeneic HSCT donor–recipient pairs (2 related pairs and 14 unrelated pairs) after DNA extraction from mononuclear cells’ fraction of PB or BM. The results confirmed the high informativity of Indel markers, which can also be used for chimerism detection.

In sum, due to its high sensitivity and drawbacks, some scholars consider that the qPCR is more suitable to be a complementary tool for the standard STR-PCR method rather than its replacement [43,55].

### 3.3. Chimerism Monitoring through Digital PCR (dPCR)

This technique has been described by different authors and consists of partitioning the whole PCR mix into thousands of droplets which act as independent “mini-reactors”. As a consequence, the DNA template becomes diluted, resulting in different compartments with and without the template. DNA templates are amplified separately among each other during the PCR phase, and the amplification result is then evaluated through fluorescence-detection techniques. At this point, both positive and negative “mini-reactors” can be counted. These authors claimed that one of the main advantages of dPCR is that it does not require the use of replicates given that each droplet can be considered to be a separate reaction [26,31,37,58].

The markers which are commonly analyzed by dPCR are Indels, SNPs, copy number variations, or Human Leukocyte Antigen (HLA)-disparities [31]. In particular, some authors claim that dPCR is able to absolutely quantify a target DNA sequence with higher precision compared with qPCR and without requiring any calibration curve [12,26,53]. In accordance with Waterhouse et al. [12], dPCR can be used as a surrogate indicator of MRD in the hematological field. In fact, chimerism analysis is able to identify donor and recipient cells but not neoplastic cells. When a neoplastic cell-specific marker is available, it should be used to analyze MRD in combination with chimerism analysis. When no MRD specific markers are available for monitoring, chimerism analysis could be used as a surrogate approach for MRD in the assumption that, in certain clinical situations, the recipient cells detected might be neoplastic cells.

As described by Sthal et al. [26], the key advantage of the dPCR is that it results from a combination between STR-PCR and qPCR. Indeed, this technique is characterized by high accuracy and reproducibility as STR-PCR and by an enhanced sensitivity as qPCR. Therefore, some scholars agree that dPCR represents a promising technology for chimerism monitoring [49,53].

The study of Santurtún et al. [59] analyzed a total of 25 post-transplantation samples which were analyzed using three different techniques to compare the performance of dPCR with other less recent techniques (e.g., STR-PCR and Indel-PCR; the second one is similar to the first but it analyzes 38 Indel markers in a single genotyping reaction). As regards dPCR analysis, the two most informative Indels were selected. All the markers were studied using the most suitable technique after DNA isolation from PB, and the respective chimerism percentages were calculated. The authors demonstrated the existence of a strict correlation between the three techniques. Nonetheless, the dPCR was proved to be a more efficient tool to study MC because of its higher sensitivity as compared to the other technologies.

The dPCR is thus considered a rapid and sensitive technique which enables the detection of chimerism with a high sensitivity ranging from around 0.01% to 0.1% [31,38,49]. However, the dPCR is characterized by four main drawbacks. First, Santurtún et al. [59] stated that the markers usually analyzed through dPCR are bi-allelic, thus implying a low capacity of differentiation, as stated previously. They considered it necessary to perform a pre-selection step through conventional PCR before proceeding with the dPCR to evaluate which Indels are the informative ones. Secondly, despite standard procedures requiring a small amount of DNA, sensitivity could sometimes need to be improved using a larger DNA amount. However, Stahl et al. [26,53] stated that an excessive amount of DNA template could interfere with correct quantification given that all droplets become positive due to the saturation of the system. Therefore, limitations exist with regard to the maximal amount of DNA, which can be analyzed in a single dPCR reaction. Currently, some scholars consider dPCR to be quite expensive because the cost per well is higher than that of STR-PCR, although the automatization of dPCR could allow for the overcoming of this problem. Similarly, they also argued that the tuning of a multiplex reaction mode could decrease both the cost and the time of the analysis [12,53,58]. Different studies suggest that low chimerism levels can persist for a long time without any clinical manifestation. Therefore, some scholars do not agree on the importance of a higher sensitivity from a clinical point of view [12,49,58]. Furthermore, in accordance with Stahl et al. [26], high sensitivity can sometimes cause false positive results because it may lead to confusion between contamination from a recipient’s skin/epithelial cells, which can occur during the post-transplantation sample collection, and a real MC.

Overall, other studies point out that the drawbacks of dPCR account for its still rare usage in chimerism analyses. Nevertheless, they affirm that, due to its rigor, sensitivity, accuracy, and reproducibility at all levels of MC, dPCR represents a promising tool with a great chance to be used more widely in the diagnostic field for chimerism monitoring in the future [12,26,49,53].

### 3.4. Chimerism Monitoring through NGS

NGS enables the simultaneous sequencing of millions of small DNA fragments with a very high sensitivity (0.01–1%). Sequenced fragments (called “reads”) are assembled all together by mapping them onto the human reference genome through the help of sophisticated bioinformatic tools. Depending on the final purpose (e.g., sequencing of one single gene, sequencing of the whole exome, or sequencing of the entire genome), some NGS platforms are more suitable than others due to their different characteristics. Despite SNP being bi-allelic markers, given that NGS can work in multiplex, a large number of SNPs can be studied simultaneously. Some authors believe that, over the last few years, NGS platforms have turned out to be the most promising technologies in the genomic field, and their applications have been extended to different contexts, such as clinical diagnostics and other medical fields [33,45]. In the context of chimerism monitoring, the ability to work with multiplex provides an unprecedented opportunity to distinguish between two different genomes, both in the absence of pre-transplantation donor DNA and in the presence of some chromosomal abnormalities [31,60]. In chimerism studies, different panels of SNP markers were initially developed to be analyzed through NGS. However, Vives et al. [47] proved that STR and Indel markers can also be analyzed with these new high-throughput sequencing technologies to detect chimerism. In accordance with Pettersson et al. [10], when SNP or Indel panels are used, NGS cannot be used as well in patients who were transplanted with two donors.

The sensitivity and, in turn, both the detection and the quantification of low levels of DNA are influenced by the sequencing depth and by the quantity and the quality of samples, as stated by Aloisio et al. [33] and Vives et al. [47]. In particular, given that more reads are required to achieve a reliable result when the amount of target DNA is low and that NGS is characterized by a high sensitivity and a high accuracy, Aloisio et al. [33] and Vives et al. [47] also stated that NGS distinguishes itself for its higher sequencing depth. In addition, as suggested by Lee et al. [60], NGS technology results improved several aspects which permitted the overcoming of the various technical limitations of the STR-PCR (e.g., stutter peaks and allele imbalance). They also argue that NGS is more and more used to perform simultaneous monitoring of MRD and chimerism. This possibility offered by NGS represents a great advantage because it guarantees an earlier detection of chimerism, often even before its clinical manifestation. Indeed, after allogeneic HSCT, donor cell-derived hematologic malignancies could sometimes represent a complication whose diagnosis can be delayed until blasts emerge in PB, and this can happen given that a CC instead of an MC appears [60].

Different authors suggested that NGS represents an innovative technology but with some technical limitations, such as a lack of standardization, high background error rate, repetitive amplification of the same reads, relatively long analysis time, and high infrastructure costs. Due to these limitations, more research is necessary before its routine application in chimerism monitoring. Moreover, these authors believe that the long time and the high cost of analysis represent the two most important limits to adopting NGS technologies in clinical field [31,60,61].

In sum, some works agree that NGS is a sensitive, accurate, and promising technology but one that is also too complex, expensive, and immature to gain a central role in chimerism monitoring. However, they consider that NGS automatization as well as the continuous reduction cost per NGS run could lead to the integration in laboratories of NGS-based methods for chimerism quantification over the next few years [31,33,60,61].

**Table 1:** Comparison among the molecular biology techniques currently available for chimerism monitoring: short tandem repeats (STR)-PCR [18,26,29,31,34,35,36,37,41,43,47,49,50,53]; quantitative PCR (qPCR) [9,17,18,31,34,38,47,49,50,55]; digital PCR (dPCR) [12,26,31,38,40,47,49,53,58]; and NGS [10,31,33,45,47,60,61].

Technique	Advantages	Technical Limitations	Input DNA Needed (ng)	Sensitivity
STR-PCR	Pre-transplantation donor and recipient samples not always necessary; Capability of also differentiating individuals in multi-donor transplantation; STR markers have a good informative rate; Simultaneous amplification of the same markers for both donor and recipient; Broad range of chimerism monitoring; Simple and universal.	Stutter peaks, pull-up peaks, or off-scale peaks interference; Amplification bias due to preferential amplification of the smallest alleles; Influenced by microsatellite instability (MSI) events.	1 ng	Sensitivity of 1%
qPCR	Easy, sensitive and fast.	Bi-allelic polymorphisms (SNPs or Indels) determine a low power of differentiation; No differentiation between individuals in multi-donor transplantation; False positive results due to its sensitivity; Range of chimerism monitoring with a lower upper limit (approximately of 30%); Necessity of creating calibration curves using donor and recipient’s pre-transplantation samples; Results can be affected by PCR inhibitors and background signals; Expensive technique; Necessity of duplicates.	From 100 pg to 1 μg	Sensitivity ranging from 0.01% to 0.1%
dPCR	Absolute quantification of the target; Calibration curves not required; Allows for the simultaneous study of minimal residual disease (MRD) and chimerism; High accuracy and reproducibility; Replicates not required; Rapid and accurate; Small DNA amount requested; Multiplex working mode is possible; Automatable.	Bi-allelic polymorphisms (SNPs or Indels) determine a low power of differentiation; Limited DNA amount in a single reaction; Expensive technique; False positive results due to its sensitivity; Still rarely used in clinical diagnostics.	400 ng	Sensitivity ranging from 0.01% to 0.1%
NGS	It can analyse STRs, Indel, or SNPs. Hhowever, given that it works in multiplex, bi-allelic markers also guarantee a high power of discrimination also when pre-transplantation donor DNA is lacking or chromosomal abnormalities are present; High depth of coverage (generally); Provides high amount of high-quality data; Allows for the simultaneous study of MRD and chimerism; Automatable; Accurate.	Lack of standardization; High background error rate; Repetitive amplification of the same reads; Relatively long analysis time; Laborious and expensive.	10 ng	Sensitivity ranging from 0.01% to 1%

## 4. Discussion

Allogeneic HSCT represents a curative option for several hematologic diseases. Chimerism analysis is important for monitoring the engraftment of donor cells within the recipient. In particular, chimerism is expressed as the percentage ratio between the number of donor and recipient cells. A successful transplantation should result in complete chimerism. However, if this happens at an early stage, the CC can result in GVHD, whereas a progressive MC seems to be connected to the rejection of the transplantation or the recurrence of disease [7,12,49,55].

Different aspects of the techniques described in this review are discussed regarding the complexity of the technique itself, their sensitivity, the availability of pre-transplantation DNA, the amount of DNA template, and the possible presence of artefacts.

Currently, STR-PCR analysis represents the gold standard to systematically monitor chimerism in patients after allogeneic HSCT. Recently, the landscape of molecular biology technologies available for this diagnostic procedure has expanded [31,34,38,47,49].

Each technique features intrinsic characteristics influenced by both instrumentation and polymorphisms which are used. STR-PCR is a rapid, simple, and universal technique which analyzes STR markers ensuring a high power of differentiation between individuals. This typical characteristics of multi-allelic STR markers allows for a reliable monitoring of multi-donor transplantation in which the probability of finding shared alleles is greater [29]. However, STRs can sometimes be affected by MSI events, which can hamper analysis if considered in the chimerism calculation [54]. Conversely, qPCR is quite simple and fast, but it is based on SNP or Indel marker detection. Given that SNP and Indel are bi-allelic markers with a lower power of differentiation in comparison with STRs, qPCR is not useful for chimerism monitoring in the case of multiple-donor transplantation [9,31,55]. Similarly to qPCR, dPCR analyses both SNP and Indel markers, but it also investigates the STR polymorphisms the and HLA-disparities [31]. Although bi-allelic markers are analyzed, the multiplex mode of dPCR assays allows for the overcoming of the limited information power typical of these DNA polymorphisms [12,53,58]. The most recent NGS technology can use SNP, STR, or Indel markers according to the chosen panel, resulting in a different informative rate. Moreover, the ability to work in multiplex reduces the drawbacks which can be attributed to the bi-allelic markers in this case as well [10,45,47,60].

Besides increasing the capability of differentiation of bi-allelic markers in dPCR and NGS, multiplex analysis decreases both the consumption of DNA and the time of analysis when multiple markers are evaluated. In particular, several studies performed an STR-PCR optimization in order to select the most advantageous markers for a better multiplex chimerism analysis [29,41,52]. Moreover, in contrast to qPCR, which separately analyzes donor and recipient markers, STR-PCR has the advantage of performing a simultaneous processing of the various markers in the same reaction tube [47,50,56]. Conversely, given that, in qPCR, donor and recipient markers are studied separately and that qPCR is characterized by a low upper bound of detection, a second analysis with the donor’s markers could be required when the level of MC increases beyond a certain threshold [17,56].

In general, the sensitivity of the various techniques that have been considered in this review highly influences chimerism detection and hence also the beginning of therapeutic intervention [33,47]. Different studies suggest a lineage-specific selection process to increase the sensitivity of the analysis. Nevertheless, in order to be considered a valid alternative, the higher informative rate should balance the increase of both the costs and the time of analysis [12,34,40].

STR-PCR and dPCR are able to detect high levels of MC, while qPCR has a low upper bound of detection above which a correct MC detection is not guaranteed [17,50,55]. In regard to NGS, its high sensitivity and accuracy are possible thanks to the high sequencing depth and the sample quality and quantity which characterize the assay. Despite all of this, doubts exist that a higher sensitivity should necessarily have a clinical significance in terms of therapeutic strategies or prognostic value [49,50]. In addition, with more sensitive techniques, we have to consider that false positive MC can be due to skin, epithelium, or stromal recipient cells collected together with PB and BM samples [26,34], which deserve attention and further studies to explore whether qPCR, dPCR, and NGS can enable an earlier therapeutic intervention [30,38,49,60].

The properties of each technique and the informative rate associated with the different polymorphisms can also influence the need for pre-transplantation samples. In the case of STR-PCR, the lack of donor and recipient pre-transplantation DNA samples seems not to be a real problem given that several solutions for STR-PCR follow-up exist [34,51]. In fact, under these circumstances, the buccal swabs may be collected after transplantation from both donor and recipient, and the two STR profiles are then obtained for the comparison of the results. In contrast to STR-PCR, in the case of qPCR, the lack of pre-transplantation samples interferes with the follow-up given that pre-transplantation samples are necessarily required to generate calibration curves in each assay [34]. Similarly to STR-PCR, both dPCR and NGS technologies do not require pre-transplantation DNA to generate calibration curves for each assay. Furthermore, dPCR does not need to use replicates because its partitioning of the DNA sample generates “mini-reactors” which can be considered independent units [26,31,38,58]. NGS is characterized by the exceptional capacity to distinguish between two different genomes, even in the absence of pre-transplantation donor DNA or in the presence of chromosomal abnormalities [30,60].

In addition to the availability of pre-transplantation DNA samples, qPCR requires large amounts of recipient pre-transplantation DNA template to guarantee the repetition of the analysis of the pre-transplantation DNA samples in each assay [34].

Biological and/or instrumental artifacts are drawbacks common to all the analyzed techniques. The main parameters which influence STR-PCR chimerism monitoring are stutter peaks and allele imbalances, which are determined by the preferential amplification of the smaller alleles, as well as pull-up peaks and off-scale peaks, which can interfere with chimerism quantification, altering the peak areas. In chimerism calculation, the STR alleles which are influenced by these alterations are excluded in order to reach a reliable chimerism result. These typical aspects of STR markers analyzed in capillary electrophoresis coupled with fluorescence detection cannot occur in qPCR, dPCR, or NGS [18,34,35,36,41]. This certainly does not mean that the other methods do not present technical limitations. Indeed, qPCR chimerism detection requires a labor-intensive optimization and can be easily influenced by background signal and by PCR inhibitors [30,56]. dPCR, as already mentioned, is strictly dependent on DNA concentration, which cannot exceed a certain threshold [26,53]. Finally, NGS lacks a standardized protocol, requires powerful bioinformatics tools and experts for the analysis, and has the highest infrastructure costs [31].

Several works suggest that chimerism analysis and MRD monitoring may result in an improved chimerism characterization, although in this case, it needs a sensitivity of at least 0.1% in order for it to be possible. For this reason, the STR-PCR could not be considered an appropriate technique [55]. Conversely, qPCR, dPCR, and NGS can be used to analyze not only the chimerism but also MRD in particular; dPCR enables multiplex, absolute quantification of target and endogenous genes simultaneously with a higher precision [12,26,53]. In NGS, the simultaneous monitoring of MRD and chimerism is feasible due to the fact that it may allow for the detection of low amounts of recipient cells, even if the clinical meaning of this earlier chimerism detection still has to be explored in greater depth [60].

Against this background, evidence for an absolutely perfect technique, which could be optimal in every situation, is currently lacking. STR-PCR is now the most applied technique in routine monitoring of chimerism after allogeneic HSCT, although it has the limit of a lower sensitivity in comparison with the other mentioned techniques. The widespread use of STR-PCR is due to the fact that it is a fast and universal technique but, above all, because of its high informative rate associated with the STR markers [17,18,26,38,43,53].

In order to overcome the limit of sensitivity, qPCR was the first to be developed. This technology ended up not being very potent except for its high sensitivity. Indeed, the high amount of input DNA required, the need for calibration curves, the low discrimination power of bi-allelic markers, and the low number of analyzable variants make the qPCR a complementary tool to anticipate chimerism detection rather than a replacement for STR-PCR [43,55].

dPCR is still rarely adopted but could represent the future of chimerism monitoring, given that it combines the advantages of both STR-PCR and qPCR (i.e., high accuracy and reproducibility and enhanced sensitivity) [12,26,49,53].

NGS can only be used as a supplement for chimerism monitoring because it lacks shared experimental protocols, and it is quite complex, time-consuming, and expensive. However, despite these problems, it seems to be a very promising technique due to its high-throughput sequencing power to detect chimerism [10,31,33,60,61].

## 5. Conclusions

In conclusion, STR-PCR today remains the most widely used method among the different molecular biology techniques proposed for chimerism monitoring. However, in comparison with both qPCR and NGS, dPCR represents a very promising alternative technology to replace STR-PCR over the short term thanks to its high sensitivity and flexibility. However, NGS is also expected to be a promising technique which will replace the previous methods once a shared experimental protocol exists and the time and the cost of the analysis are reduced. Eventually, further studies aimed at optimizing these techniques are particularly welcome, considering that a greater technical ability to differentiate among different individuals or sources can have important implications in different clinical and medical fields.

## Data Availability

The data presented in this study are available on request from the corresponding author.
